# A rapid thioacidolysis method for biomass lignin composition and tricin analysis

**DOI:** 10.1186/s13068-020-01865-y

**Published:** 2021-01-11

**Authors:** Fang Chen, Chunliu Zhuo, Xirong Xiao, Thomas H. Pendergast, Katrien M. Devos

**Affiliations:** 1 BioDiscovery Institute and Department of Biological Sciences, University of North Texas, 1155 Union Circle #311428, Denton, TX 76203 USA; 2 Institute of Plant Breeding, Genetics and Genomics, Department of Crop and Soil Sciences, and Department of Plant Biology, University of Georgia, Athens, GA 30602 USA; 3 Center for Bioenergy Innovation (CBI), Oak Ridge National Laboratory, Oak Ridge, TN 37831 USA

**Keywords:** Biomass, Lignin, Tricin, Thioacidolysis, High throughput

## Abstract

**Background:**

Biomass composition varies from plant to plant and greatly affects biomass utilization. Lignin is a heterogeneous phenolic polymer derived mainly from *p*-coumaryl, coniferyl, and sinapyl alcohols and makes up to 10–25% of lignocellulosic biomass. Recently, tricin, an *O*-methylated flavone, was identified as a lignin monomer in many grass species. Tricin may function as a nucleation site for lignification and is advocated as a novel target for lignin engineering to reduce lignin content and improve biomass digestibility in grasses. Thioacidolysis is an analytical method that can be adapted to analyze both lignin monomeric composition and tricin content in the lignin polymer. However, the original thioacidolysis procedure is complex, laborious, and time consuming, making it difficult to be adopted for large-scale screening in biomass research. In this study, a modified, rapid higher throughput thioacidolysis method was developed.

**Results:**

In combination with gas chromatography–mass spectrometry (GC–MS) and liquid chromatography–mass spectrometry (LC–MS), the modified thioacidolysis method can be used to simultaneously characterize the lignin composition and tricin content using 2–5 mg of dry samples. The modified method eliminates the solvent extraction and drastically improves the throughput; 80 samples can be processed in one day per person. Our results indicate that there is no significant difference in the determination of lignin S/G ratio and tricin content between the original and modified methods.

**Conclusions:**

A modified thioacidolysis protocol was established. The results demonstrate that the modified method can be used for rapid, high-throughput, and reliable lignin composition and tricin content analyses for screening transgenic plants for cell wall modifications or in large-scale genome-wide association studies (GWAS).

## Background

Plant biomass is an abundant renewable feedstock that can be converted to liquid transportation fuels by fermentation. Although lignin negatively affects cell wall saccharification, it also has potential as a natural source for high-value products [1–3]. Lignins are complex natural polymers resulting from oxidative coupling of 4-hydroxycinnamyl alcohols. Their composition and structure vary by plant species, tissue, cell type, and developmental stage. Lignin polymers are formed by combinatorial phenolic radial coupling reactions catalyzed by laccases and/or peroxidases. As a result, the lignin molecules are heterogeneous in composition, molecular weight, cross linking, and functional groups [4]. In plant biomass, lignin composition is determined by the content of its subunits, that is, *p*-hydroxyphenyl-(H), guaiacyl-(G), and syringyl-(S) units, derived from *p*-coumaryl alcohol, coniferyl alcohol, and sinapyl alcohol, respectively. In lignin polymers, these subunits are attached to one another by a series of characteristic linkages (*β*-O-4, *β*-5, *β*-*β*, etc*.*) [5, 6]. Recently, non-conventional monomers, such as tricin, piceatannol, feruloyltyramine, and caffeyl alcohol, were shown to also be authentic subunits of lignin [7–11]. Due to its tight association with other cell wall component, it is impossible to isolate lignin from the plant cell wall without altering its structure, and determination of the lignin structure in vivo remains a great challenge. There is no single analytical method that provides fast, conclusive lignin characterization [12]. Several analytical methods have been used to characterize the non-conventional units integrated in lignin polymers [9, 13, 14], but high-throughput methods for quantification of these lignin components are still lacking.

Thioacidolysis is one of the most widely utilized methods for lignin structure and compositional analysis [15–21]. It has also been used to characterize the non-conventional lignin units, such as tricin in plant biomass [22]. Thioacidolysis releases the monomers that are only involved in *β*-O-4 aryl ether linkages. Other linkages can be determined by analysis of dimers by gas chromatography–mass spectrometry (GC–MS) after thioacidolysis followed by Raney nickel desulfurization [15, 16]. Thioacidolysis is routinely used to estimate the amount and composition of uncondensed aryl ether structures in lignin. However, this method uses malodorous ethanethiol and the experimental procedure is laborious and time consuming, rendering it undesirable for use by many researchers, especially when a larger number of samples need to be analyzed. Several streamlined thioacidolysis procedures have been developed to improve the throughput [23–25]. However, all protocols still involve a tedious solvent extraction component that severely limits their wide adoption.

Here we report a simplified process that completely eliminates the solvent extraction. The modified method can be used to determine lignin composition and tricin content in various types of biomass. The simple experimental set-up allows routine analysis of 80 samples per day and can be adopted by most biological labs, including those lacking sophisticated analytical set-ups and instruments.

## Results

### A modified thioacidolysis method for lignin composition screening

**Fig. 1:**
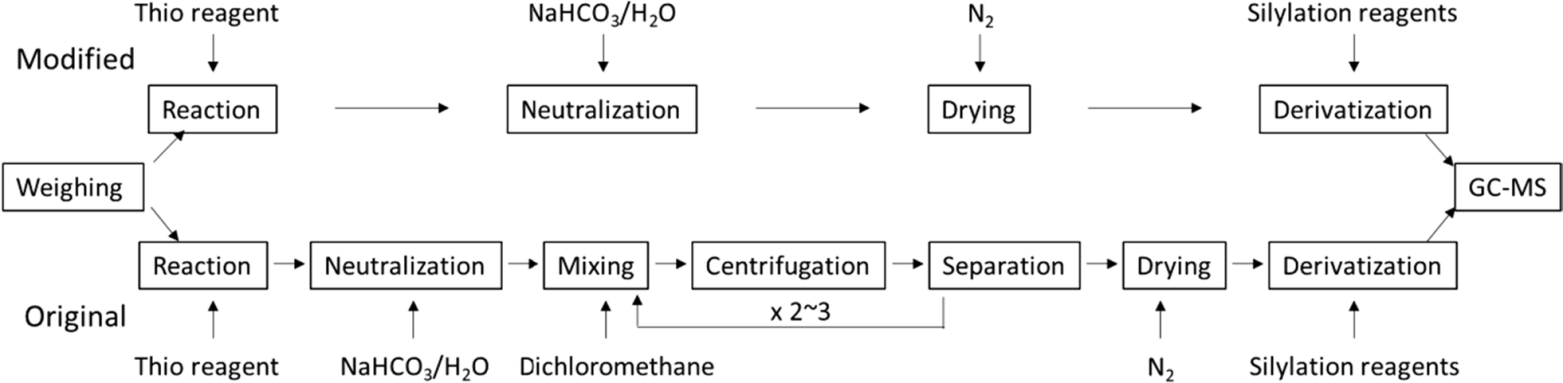
A workflow chart of the original and modified thioacidolysis procedures. Removal of the solvent–solvent extraction step from the procedure reduces the time of sample processing and eliminates the use of the hazardous chemical dichloromethane

**Fig. 2:**
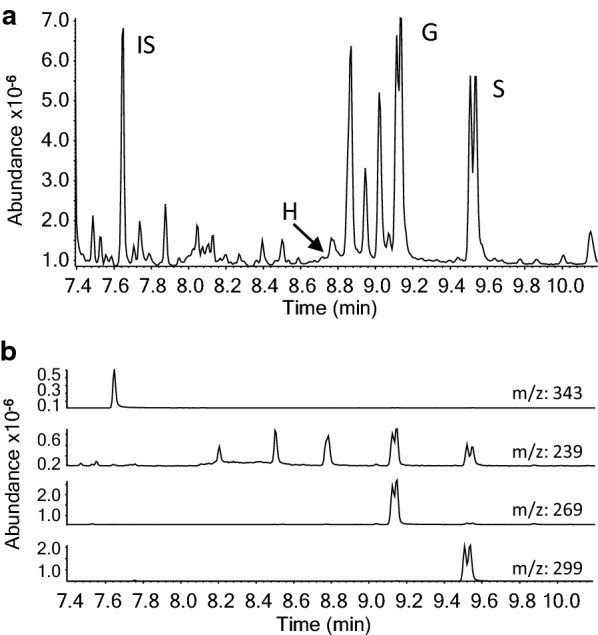
A representative GC–MS chromatogram of TMS derivatives of bagasse thioacidolysis products prepared by the modified method. **a** A total ion chromatogram. The double peaks of G and S represent the *erythro* and *threo* isomers of guaiacyl and syringyl units released by thioacidolysis. **b** Selected ion chromatograms for *m/z* 343 (IS), 239 (H monomer), 269 (G monomer), and 299 (S monomer). IS: Internal standard; H: *p*-hydroxyphenyl monomer; G: Guaiacyl monomer; S: Syringyl monomer

A simple equipment set-up comprising a semi-automated single syringe dispenser (Hamilton Co., USA) and an 80-position high-temperature dry block/nitrogen evaporator (Organomation Associates Inc. USA) was used to accurately deliver the reaction reagents and improve the throughput of the analysis (Additional file 1). The equipment was placed in a standard chemical hood so that all steps of the thioacidolysis protocol could be carried out within the hood. Figure 1 shows the workflow of the original and modified procedures. In the original thioacidolysis protocol, reaction mixtures were first neutralized with sodium bicarbonate solution and then extracted with dichloromethane. The extracted thioacidolysis products were derivatized with silylation reagent before subjecting them to gas chromatography. To improve the throughput of the analysis, we investigated if the solvent extraction step could be eliminated. Direct drying of the reaction mixture followed by derivatization with silylation reagent was unsuccessful due to breakdown of the reaction products during the drying process. However, if the reaction mixture was first neutralized with aqueous sodium bicarbonate and then dried under a stream of nitrogen, the dried product residual could be derivatized with N,O-bis(trimethylsilyl)trifluoroacetamide (BSTFA). It appears that the generated salts after neutralization (NaBF_4_, Na_2_B_4_O_7_) were inert and did not interfere with the silylation process. Elimination of the solvent–solvent extraction significantly reduced both the sample processing time and the chance of accidentally releasing malodorous ethanethiol to the laboratory environment. With the new equipment set-up and modified protocol, we were able to process up to 80 samples at a time, up from two dozen samples per day with the original method. Figure 2 shows a typical GC–MS chromatogram of the trimethylsilyl (TMS) derivatives of thioacidolysis products from a bagasse sample. The double peaks of *erythro* and *threo* isomers of guaiacyl and syringyl unit-derived monomers are characteristic for lignin thioacidolysis products.

**Fig. 3:**
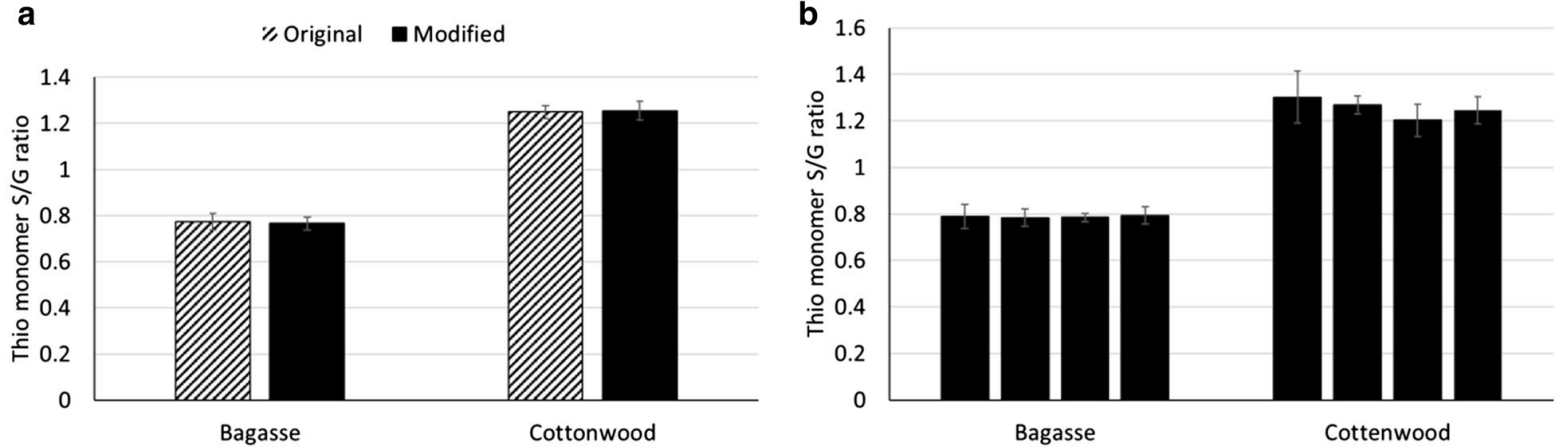
Lignin composition of bagasse and cottonwood samples. a. Average lignin S/G ratios measured by original and modified thioacidolysis (*n* = 8). Values obtained with the two methods are not statistically different (*p* > .05); b. Average Lignin S/G ratios measured by the modified method of the same samples (*n* = 6) in four different experiments. No significant differences were observed between experiments (*p* > .05)

To validate the modified protocol, we analyzed bagasse and cottonwood reference materials obtained from the National Institute of Standards and Technology with the original method with solvent extraction and with the modified method. As shown in Fig. 3a, there is no significant difference in the determined lignin S/G ratios for either material when analyzed by these two methods. The standard deviations of the S/G ratios for all samples were around 5% for both methods. To check the reproducibility of the modified method, we analyzed the reference materials using the modified method in four batches. There were no significant differences in the lignin S/G ratio within bagasse or cottonwood samples among the four batches (Fig. 3b).

**Fig. 4:**
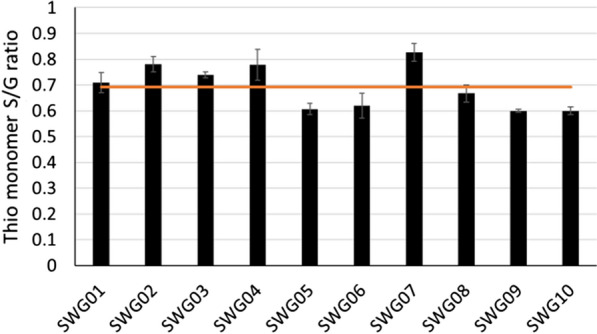
Variation in lignin S/G ratio of 10 F_2_ lines derived from a cross between a switchgrass lowland ecotype with an upland ecotype. The horizontal line represents the average S/G ratio of the 10 samples (*n* = 3). The error bar shows the standard deviation of triplicate analyses of individual samples

**Fig. 5:**
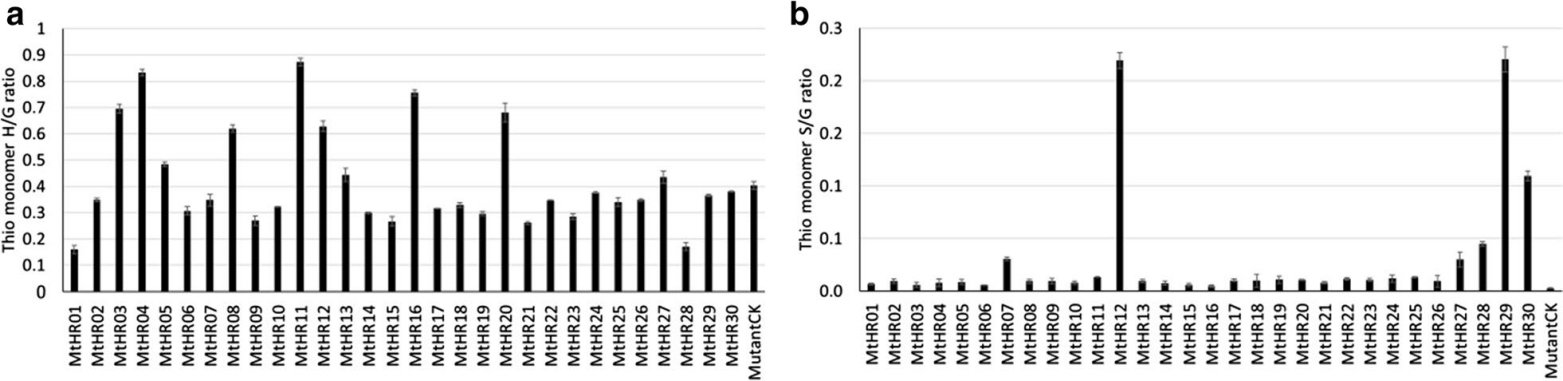
Lignin monomer composition of transgenic hairy roots derived from *comt* mutants of *Medicago truncatula* (*n* = 2). **a** Monomer H/G ratio; **b** Monomer S/G ratio. Knockout of the *COMT* gene blocked S lignin biosynthesis. However, S lignin biosynthesis was reinstated in some of these transgenic lines

The modified method was then used to analyze the variation in lignin S/G ratio present in F_2_ switchgrass progeny derived from a cross between a lowland and an upland ecotype. The S/G ratios (0.6–0.8) are comparable with previously published data and show a strong correlation with results obtained by Pyrolysis Molecular Beam Mass Spectroscopy [25–28]. The average standard deviation of triplicate analyses of each sample (4%) is significantly smaller than the variation of the S/G ratio within the group of samples (12%) (Fig. 4), demonstrating the suitability of the method for analyzing the variation in lignin composition present in segregating or natural populations. We also used our new protocol to analyze two replicates each of 30 transgenic *Medicago truncatula* hairy root samples. Our data clearly show that manipulation of lignin pathway genes in *M. truncatula* significantly changed the lignin composition in hairy roots (Fig. 5).

### Thioacidolysis monomer yield of the modified method

**Fig. 6:**
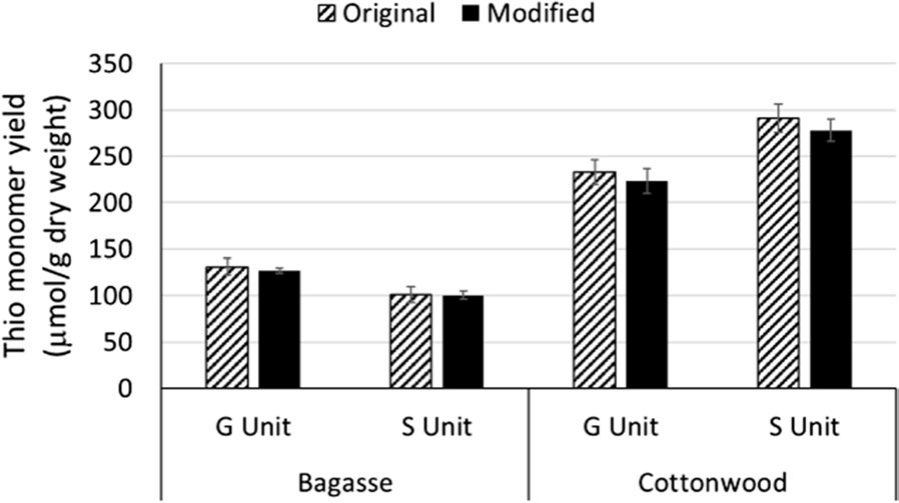
Thioacidolysis monomer yields measured by the original and modified methods. The error bar shows the standard deviation of individual samples (*n* = 8). Yields obtained with the two methods were not significantly different (*p* > .05)

Thioacidolysis monomer yield generally depends on both the lignin structure and total lignin content of the biomass. In most angiosperm plant samples, G- and S-derived monomers are the major products of thioacidolysis, while the H-derived monomers are at trace levels. Figure 6 shows the comparison of G- and S-derived monomer yields of bagasse and cottonwood samples measured by the original and modified methods. The amount of variation seen in monomer yields is similar with both methods. Similarly, there is no significant monomer yield difference between the two methods for bagasse and a slight, but non-significant, decrease in monomer yield in cottonwood samples with the modified compared to the original method. The decrease in monomer yield in cottonwood can be seen for both the G- and S-derived monomers (Fig. 6). The level of change does not appear to favor one monomer over another, leading to very similar S/G ratios for both analytical methods (Fig. 3a).

### Modified thioacidolysis method for tricin content analysis

Tricin is now considered to be a genuine monomer of lignin in monocots, and in some dicots. Tricin in free or conjugated form can be extracted and quantified by high-performance liquid chromatography (HPLC) [29–31]. To characterize and quantify the proportion of tricin units integrated in native lignin polymers, nuclear magnetic resonance spectroscopy (NMR) or chemical degradation followed by chromatographic analysis is frequently applied [9, 10, 14]. It has been demonstrated that thioacidolysis is one of the best methods to determine tricin content in the lignin polymer, because it not only cleaves the tricin–lignin ether bond with high yields but also results in less degradation of the released tricin [22].

**Fig. 7:**
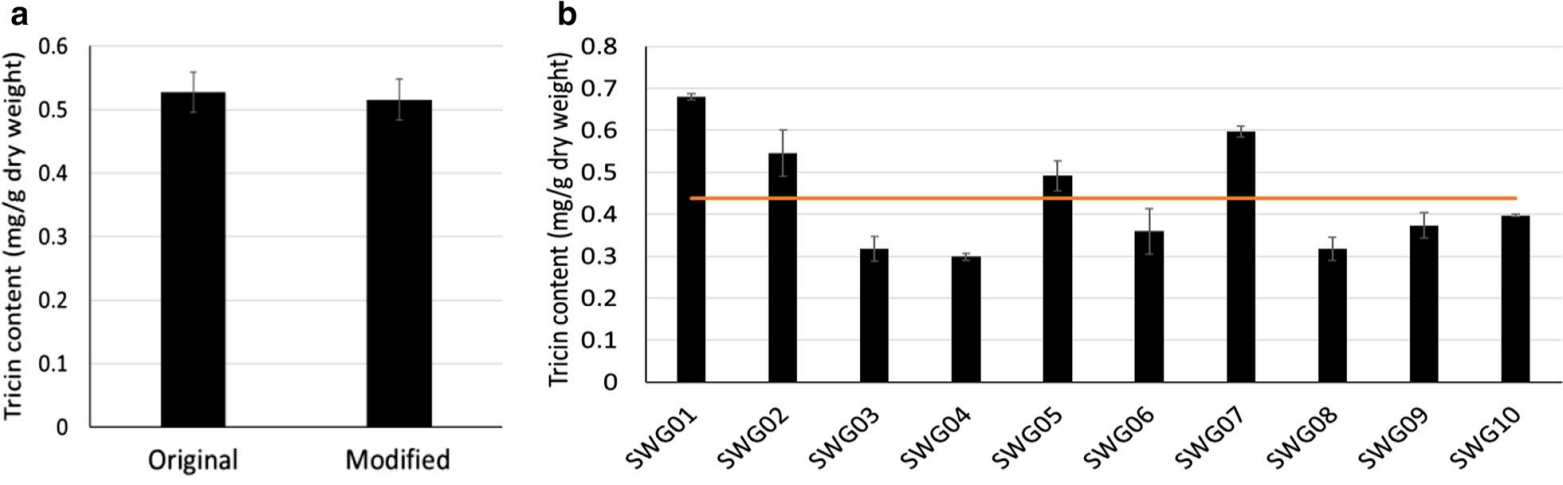
Tricin content in bagasse and switchgrass biomass. **a** Tricin content in bagasse measured by original and modified thioacidolysis. The error bar shows the standard deviation of individual samples (*n* = 8). The results were not significantly different (*p* > .05). **b** Variation in tricin content in 10 F_2_ lines derived from a cross between two switchgrass ecotypes. The horizontal line represents the average tricin content of the 10 samples. The error bar shows the standard deviation of individual samples (*n* = 3)

Here we compared the tricin content in bagasse samples using the original and modified thioacidolysis methods. There was no significant difference in tricin content detected by these two methods (Fig. 7a). As an example of application, the modified method was used to determine the variation in tricin content in a random sample of 10 progeny from a switchgrass F_2_ population. As shown in Fig. 7b, the standard deviation of technical replicates is below 4%, much smaller than the variation of tricin content within the F_2_ samples (> 30%).

## Discussion

Its high sensitivity to detect lignin and the robustness of the process make thioacidolysis the preferred choice over other degradative analytical methods to determine lignin composition. However, the original thioacidolysis procedure is laborious and time consuming. Several attempts have been made over the years to simplify and streamline the process. In one reported thioacidolysis procedure, the reaction volume was scaled down to 1 mL, and the quenching of the reaction and extraction of the products were done in the same 5-mL reaction tube [23]. The results showed that there was no significant difference in monomer ratios measured by the original and scaled-down protocols, despite the sample to solvent ratio being reduced from 1:1 (mg/mL) to 1:0.1 (mg/mL). Harman-Ware and colleagues used 2 mg of biomass with 1 mL of thio solvent and conducted the reaction in a 2-mL screw-cap vial [25]. After cooling, part of the reaction solution was transferred to another culture tube, neutralized, and extracted with ethyl acetate. The standard deviation of 10 technical replicates of a single poplar sample was less than 2% and the results were very similar to those obtained by original methods [25]. A microscale thioacidolysis method for the rapid analysis of *β*-O-4 substructures in lignin has also been reported using 5 mg of sample and 0.9 mL of solvent [24]. These reports all showed that a scaled-down reaction is possible for biomass thioacidolysis without affecting the results of the lignin compositional analysis. In our study, we chose to use 2–5 mg of sample with 1 mL of thio reagent after considering the weighing accuracy, sample homogeneity, and solvent handling. The results show that there is no significant difference in the determined lignin S/G ratios when analyzed by the original method and our modified method.

Thioacidolysis can selectively and efficiently cleave the β-O-4 linkages in the lignin polymer. Complete cleavage of *β*-O-4 linkages requires sufficient thio reagent to be present during the reaction time. The original protocol suggested a minimum of 0.5 mL of reagent per mg of tissue [16]. In a revised protocol, the solvent volume to sample weight ratio was decreased tenfold. This change caused an approximately 5% decrease in yield for both G- and S-derived monomers [23]. Our results indicate that the use of 0.25 to 0.5 mL of reagent per mg of tissue does not lead to a decrease of total monomer yield in bagasse standard materials. For cottonwood samples, a slight albeit non-significant decrease (~ 4.5%) in the yield of both G- and S-derived monomers was observed. This decrease could be due to the incomplete derivatization of thioacidolysis monomers. There were always some salts present in the sample vials when the modified method was used and this may hinder the derivatization in some samples. We found that derivatization at 55 °C for 30 min with brief vigorous vortexing was sufficient for most samples, but a prolonged derivatization time could be beneficial for some sample types. For large-scale screening projects, we suggest including three standard samples in each batch of analyses so that lignin S/G ratios and monomer yields can be normalized across sample sets.

The absolute quantification of thioacidolysis yields depends on the accuracy of determination of the relative response factor of the analytes and the internal standards. In our study, we did not attempt to determine the accurate response factor since pure lignin-derived monomers were not available to us. However, the syntheses of lignin-derived thioacidolysis monomers and their uses as quantitation standards were published by Yue et al. [27], which makes it possible to accurately determine the lignin monomer yields for different biomass materials.

The major improvement in sample throughput was achieved by the complete removal of the solvent–solvent extraction process from our protocol, which is the most time and labor-intensive part of the original thioacidolysis procedure. This modification resulted in a drastic reduction of post-reaction sample processing time, allowing 80 samples to be processed per day per person. In our hands, the limit to throughput is now shifted to the GC–MS capability and sample weighing. Harman-Ware et al. [25] reported utilizing a low thermal mass modular accelerated column heater equipped gas chromatography instrument (LTM MACH GC) to analyze the thioacidolysis reaction products, which would further reduce the analysis time.

The lack of solvent extraction in the workflow means that “dirty” samples are being used for downstream GC–MS and LC–MS analyses. To handle the large number of “dirty” samples, we adjusted the GC–MS program used. Depending on the heating and cooling capacity of the GC oven, the total running time of one sample is about 15 min. Since most of the unknown compounds were eluted before the internal standard, which was eluted after 7 min in our case, a solvent delay of 6.5 min was set to start the mass detector. We had no difficulty separating the thioacidolysis monomeric products (Fig. 2a) using our short heating program. However, the risk of using a short program is that the internal standard peak may partially overlay with the contaminants in the dirty samples. For GC–MS, this is generally not a problem because selected ions are extracted from the total ion chromatogram and used for quantification [25] (Fig. 2b). If GC-FID is used, the GC program may need to be adjusted to ensure complete separation of the internal standard and other peaks. For LC–MS analysis, the elution stream was not directed to the mass detector until minute 5 and only a two-minute window was used to detect the internal standard and tricin which, in our case, were eluted at 5.4 and 6.5 min, respectively. Due to the reduced sample quality and higher throughput of our analysis, it is recommended to use a new GC inlet septum for every set of 80 samples, and change the GC inlet and clean the mass spectrometer ion source more frequently. Even so, in an analysis of more than 800 switchgrass samples with our modified thioacidolysis protocol, no extra instrument service was required besides these regular maintenances. Our results demonstrate that the adverse impact of the dirty samples on the instrument can be mitigated by careful selection of the downstream instrument parameters.

## Conclusions

The modified thioacidolysis protocol completely eliminates the solvent extraction process and provides similar results compared to the original method. This modified protocol can be used for rapid and high-throughput analysis of lignin composition and tricin content in biomass.

## Methods

### Chemicals

High-performance liquid chromatography (HPLC) grade methanol and chloroform and LC–MS grade acetonitrile with 0.1% formic acid (v/v) and water with 0.1% formic acid (v/v) were purchased from Thermo Fisher Scientific (Waltham, USA). 1,4-dioxane, boron trifluoride diethyl etherate, ethanethiol, pyridine, and tricin were purchased from Sigma-Aldrich (St. Louis, USA). 4,4′-Ethylidenebisphenol was purchased from TCI America (Portland, USA). BSTFA + 1% trimethylchlorosilane (TMCS) silylation reagent was purchased from Thermo Fisher Scientific (Waltham, USA).

### Plant materials

The bagasse and cottonwood reference materials were obtained from the National Institute of Standards and Technology (Gaithersburg, USA). The switchgrass samples were randomly selected from F_2_ progeny derived from an AP13 (lowland ecotype) × VS16 (upland ecotype) cross, grown at the University of Georgia’s Iron Horse Farm in Watkinsville, GA. Entire plants of each genotype were harvested 3 cm above ground level, dried at 60 °C for at least 10 days, and milled using a Wiley model 4 mill with 1-mm mesh screen. The material used here was harvested from the 2017 field season. *Medicago truncatula* hairy roots were grown on petri dishes in a growth chamber and were ground to powder in liquid nitrogen and then freeze dried overnight.

### Sample preparation

About 150 mg of ground material was added to a 4-mL glass vial. The samples were sequentially extracted with 100% methanol, 50% (v/v) methanol, and water, each twice. The extract-free plant material was freeze dried by lyophilization overnight.

### Equipment set-up

A syringe dispenser (Hamilton Co. Reno, USA. Cat. No. ML620-DIS) and an 80-position MULTIVAP Nitrogen Evaporator (Organomation Associates Inc. Berlin, USA. Cat. No. 11880) were placed in a standard chemical hood and used for thioacidolysis (Additional file 1). Reactions were carried out in sets of 2-mL Micro-Reaction Vessels (Sigma-Aldrich. St. Louis, USA. Cat. No. 27037). All thioacidolysis steps were conducted within the hood, and the instrument set-up allowed samples to remain within the hood from start to finish.

### Thioacidolysis

To prepare 250 mL of thioacidolysis reaction reagent, about 100 mL of 1,4-dioxane was added into a 250-mL volumetric flask. Then, 6.25 mL of boron trifluoride diethyl etherate and 25 mL of ethanethiol were added. 4,4′-ethylidenebisphenol and umbelliferone were added as internal standards with final concentrations of 0.012 mg/mL and 0.004 mg/mL, respectively. Additional 1,4-dioxane was added to bring the total volume to 250 mL. The thioacidolysis reaction reagent can be stored in an amber glass bottle under nitrogen for up to one month. The original thioacidolysis procedure was conducted as previously published [15, 16] except that 3 mL of thioacidolysis reagent was added to 5 mg of sample. The reaction was run at 100 °C for 4 h after which the reaction mixtures were cooled, and 1 mL of saturated NaHCO_3_ solution and 4 mL of water were added. The reaction mixtures were extracted three times with dichloromethane in the reaction tube. Detailed equipment set-up and protocol for the modified thioacidolysis method are shown in Additional file 1. Briefly, about 2.5 mg of dry plant material was accurately weighed into a 2-mL Micro-Reaction Vessel. One milliliter (1.0 mL) of thioacidolysis reagent was added to each vessel. The vials were capped tightly and put into the position holes of a pre-heated MULTIVAP high-temperature dry block. The reaction was set at 100 °C for 4 h with a brief vortex every hour. Upon completion of the reaction, the vials were placed in a rack at room temperature to cool. During the cooling time, 190 µL of saturated NaHCO_3_ solution was added to two sets of 4-mL glass vials (Thermo Fisher Scientific, Waltham, USA, Cat. No. B7800-2), one set for GC–MS lignin composition analysis and another set for LC–MS tricin analysis. Then, 400 µL of the cooled solution from the reaction vials was transferred into the 4-mL glass vials and mixed by pipetting. The 4-mL glass vials were placed in the dry block at 55 °C under nitrogen gas flow until the samples had dried completely.

### Gas chromatography–mass spectrometry (GC–MS)

One set of the dried samples was derivatized with 200 µL of derivatization reagent (1:1 pyridine and BSTFA + 1% TMCS Silylation Reagent (v/v)) at 55 °C for 30 min before being subjected to GC–MS for lignin monomer analysis. An Agilent Technologies 7890A GC system with 5975C inert XL EI/CI mass selective detector (MSD) (Agilent. Santa Clara, USA) was used. An Agilent HP-5MS column (30 m × 0.250 mm, 0.25 micro) was used to separate the lignin monomers. Helium was used as carrier gas and the gas flow rate was set at 1 mL/min. The injection volume was set at 2 µL with split (1/10) injection using an autosampler. The GC inlet temperature was 230 °C. The initial oven temperature was 100 °C, held for 2 min, and then ramped at 25 °C/min to 300 °C and held for 3 min. The MSD transfer line temperature was set at 250 °C. The MS quadrupole temperature was 150 °C and the MS source temperature was 250 °C. The electron impact ionization voltage was 70 eV. The mass detector scan range was set from 50 to 500 *m/z* with a solvent delay of 6.5 min. Selected ion monitoring (SIM) chromatograms were integrated to determine the monomer yields: 343 *m/z* for the internal standard, 239 *m/z* for H-derived monomers, 269 *m/z* for G-derived monomers, and 299 *m/z* for S-derived monomers. A one-way analysis of variance (ANOVA) was used to determine if the means of the results obtained from the original and modified methods were statistically different.

### Liquid chromatography–mass spectrometry (LC–MS)

One set of the dried samples was resuspended in 600 µL of 95% methanol before being subjecting to LC–MS for tricin quantification. An Agilent HPLC 1290 infinity II system with Agilent 6460C Triple Quadrupole LC–MS System as mass detector was used. An Agilent Zorbax Eclipse Plus C18 column (2.1 mm × 50 mm, 1.8-micro) was used to separate the compounds. The HPLC mobile phases used were 0.1% (v/v) formic acid in water (A) and 0.1% (v/v) formic acid in acetonitrile (B). The column thermostat was maintained at 30 °C with a solvent flow rate of 0.45 mL/min, and the elution gradients were 6% B for 3 min, from 6 to 95% B in 5 min, stay in 95% B for 2 min, then from 95 to 6% B in 1 min and stay in 6% B for 2 min. The total run time was 13 min. An Agilent 6460C Triple Quadrupole mass spectrometer was set to run in scan mode for compound identification, and multiple reaction monitoring (MRM) mode for tricin quantification (the precursor and product ions for umbelliferone and tricin are *m/z* 161 and 133, and *m/z* 329 and 299, respectively). Nitrogen was used as sheath gas at a flow rate of 11 L/min at 350 °C. The capillary gas temperature was 300 °C, the flow rate 10 L/min, and the Nebulizer gas pressure 45 psi. The ESI spray capillary voltage was 3500 V in negative ionization mode. The fragmentor voltage was 135 and the cell accelerator voltage was 7. Processing of data was done off-line using the Agilent Masshunter qualitative data analysis software.

## Supplementary Information

**Additional file 1.** The modified thioacidolysis protocol for rapid biomass lignin and tricin analysis.

## Data Availability

Data are available from the corresponding author on reasonable request.

## Abbreviations

BSTFA: *N*,*O*-Bis(Trimethylsilyl)trifluoroacetamide
COMT: Caffeic acid *O*-methyltransferase
GC-FID: Gas chromatography with flame ionization detection
GC–MS: Gas chromatography–mass spectrometry
GWAS: Genome-wide association study
HPLC: High-performance liquid chromatography
LC–MS: Liquid chromatography–mass spectrometry
MRM: Multiple reaction monitoring
SIM: Selected ion monitoring
TMCS: Trimethylchlorosilane
TMS: Trimethylsilyl
