# Supplementary material for: A rapid thioacidolysis method for biomass lignin composition and tricin analysis
Source: Biotechnol Biofuels. 2021 Jan 11;14:18. doi: 10.1186/s13068-020-01865-y (PMC7798261; doi:10.1186/s13068-020-01865-y)
Supplement: Supplementary file 1 — Additional file 1. The modified thioacidolysis protocol for rapid biomass lignin and tricin analysis. [file 13068_2020_1865_MOESM1_ESM.docx]

**A RAPID THIOACIDOLYSIS METHOD FOR BIOMASS LIGNIN COMPOSITION AND TRICIN ANALYSIS**

**REAGENTS:** The following reagents were used in this study. The chemicals with equivalent quality obtained from other sources can be used instead.

Milli-Q water (ultrapure water; Millipore, St. Louis, USA); Methanol, HPLC grade (Fisher Chemical, Waltham, USA. Cat. No. A452-4); Chloroform, HPLC grade (Fisher Chemical, Cat. No. C607SK-1); Acetonitrile with 0.1% Formic Acid (v/v), LC/MS Grade (Fisher Chemical, Cat. No. LS120-212); Water with 0.1% Formic Acid (v/v), LC/MS Grade (Fisher Chemical, Cat. No. LS1184); 1,4-Dioxane (Sigma-Aldrich INC, St. Louis, USA. Cat. No. 296309); Boron trifluoride diethyl etherate (Sigma-Aldrich INC, St. Louis, USA. Cat. No. 216607); Ethanethiol (Sigma-Aldrich INC, Cat. No. E3708); 4,4'-Ethylidenebisphenol (TCI America, Portland, USA. Cat. No. E0432); Pyridine (Sigma-Aldrich INC, Cat. No. 270970); BSTFA + 1% TMCS Silylation Reagent (ThermoFisher Scientific INC, Waltham, USA. Cat. No. TS-38833); Tricin (Sigma-Aldrich INC, Cat. No. PHL80987)

**EQUIPMENT:** The following equipment were used in this study. Similar equipment with equivalent function can be used instead.

Sonicator (Bransonic ultrasonic cleaner model 2510, Sigma-Aldrich INC, Cat. No. Z244910); Centrifuge (Sorvall Legend XTR Centrifuge, ThermoFisher Scientific INC, Cat. No. 75004521); Micro-Reaction Vessels (Sigma-Aldrich. St. Louis, USA. Cat. No. 2-7037); Freeze Dryer (Labconco Co, Kansas City, USA. Cat. No. 711211010); 80 Position MULTIVAP Nitrogen Evaporator (Organomation Associates Inc. Berlin, USA. Cat. No. 11880); Syringe Dispenser (Hamilton Co. Reno, USA. Cat. No. ML620-DIS); GC-MS instrument: Agilent Technologies 7890A Gas Chromatograph with 5975C iner XL EI/CI MSD detector (Agilent. Santa Clara, USA); LCMS: Agilent HPLC 1290 infinity II system with Agilent 6460C Triple Quadrupole LC/MS System as mass detector (Agilent. Santa Clara, USA).

**PLANT MATERIALS**

Plant materials can be oven-dried, air-dried or freeze-dried. Woody plants should be de-barked. For herbaceous plants, stem could be separated from leaf or whole tiller could be used. Samples can be milled using a Wiley model 4 mill with 1 mm mesh screen or ground to powder in liquid nitrogen and then freeze dried. For each analysis, about 2-5 mg of ground sample is required depending on the lignin content of the plant biomass.

**REAGENT SETUP**

Preparation of a standard stock solution of bisphenol E: Dissolve 20.0 mg of 4,4'-ethylidenebisphenol in 10 mL of 1,4-dioxane to make a stock solution of 2.0 mg/mL internal standard.

Preparation of a standard stock solution of umbelliferone: Dissolve 10.0 mg of umbelliferone in 10 mL of 1,4-dioxane to make a stock solution of 1.0 mg/mL internal standard.

Preparation of a standard to determine the response factor of tricin in LC-MS analysis: A standard containing 0.0416 mg/mL tricin and 0.0416 mg/mL umbelliferone was made by separately dissolving 1.0 mg of tricin and 1.0 mg of umbelliferone in 3 mL of methanol, diluting them four-fold and then mixing them in 1:1 ratio.

Thioacidolysis reaction reagent: In a 250 mL volumetric flask, add about 100 mL 1,4-dioxane, then add 6.25 mL of Boron trifluoride diethyl etherate, 25 mL of ethanethiol, 1.5 mL of 4,4'-ethylidenebisphenol and 1.0 mL of umbelliferone internal standard stock solution, then add 1,4-dioxane to a volume of 250 mL. The final internal standard concentration is 0.012 mg/mL of 4,4'-ethylidenebisphenol and 0.004 mg/mL of umbelliferone. The thioacidolysis reaction reagent can be stored in an amber glass bottle under nitrogen for up to one month.

Derivatization reagent: Mix 1:1 pyridine and BSTFA + 1% TMCS silylation Reagent (vol/vol) in a glass tube with screw cap.

**EQUIPMENT SET-UP**

A syringe dispenser (Hamilton Co. Cat. No. ML620-DIS) and an 80 position MULTIVAP Nitrogen Evaporator (Organomation Associates Inc. Cat. No. 11880) placed in a standard chemical hood were used for thioacidolysis. The entire thioacidolysis procedure was completed within the hood.


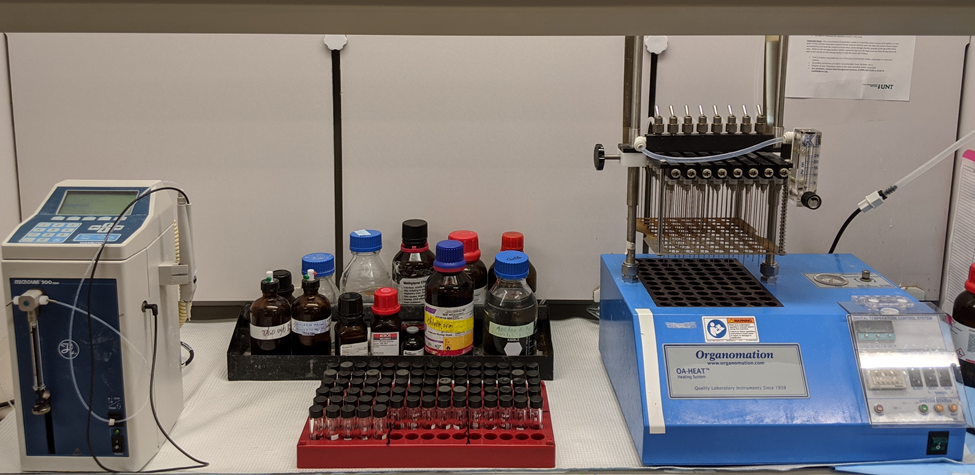


GC-MS: An Agilent Technologies 7890A GC system with 5975C MSD was used for lignin monomer analysis. Helium was used as carrier gas and the carrier gas flow rate was set at 1.0 mL/min. A HP-5MS column (30 m x 0.250 mm, 0.25-micro) was used to separate the lignin monomers. The injection volume was set at 2 μL with split (1/10) injection using an autosampler. The GC inlet temperature was 230 °C. Perform eight syringe washes with chloroform between samples. The initial oven temperature was 100 °C, held for 2 min, and then ramped at 25 °C/min to 300 °C and held for 3 min. The mass selective detector (MSD) transfer line temperature was set at 250 °C. The MS quadrupole temperature was 150 °C, the MS source was set at 250 °C, and the electron impact ionization voltage was set at 70 eV. The mass detector scan range was set from 50 to 500 m/z with a solvent delay of 6.5 min. For lignin monomer quantification, the following selected ion monitoring (SIM) chromatograms were integrated to determine monomer yields: 343 m/z for the internal standard (4,4'-ethylidenebisphenol); 239 m/z for H-derived monomers; 269 m/z for G-derived monomers; and 299 m/z for S-derived monomers. Data processing is done off-line using Agilent’s ChemSation qualitative data analysis software.

LC-MS: An Agilent HPLC 1290 infinity II system consisting of a G7129B Vialsampler, a G7104A Flexible pump, a G7116B Multicolumn Thermostat and a G7117A Diode Array Detector was used for tricin analysis. An Agilent Zorbax Eclipse Plus C18 column (2.1 X 50 mm, 1.8-micro) was used to separate the compounds. The HPLC mobile phases used were water with 0.1% (v/v) formic acid (A) and acetonitrile with 0.1% (v/v) formic acid (B). The column thermostat was maintained at 30 °C with a solvent flow rate of 0.45 ml/min, and the elution gradients were 6% B for 3 min, from 6% to 95% B in 5 min, stay in 95% B for 2 min, then from 95% to 6% B in 1 min and stay in 6% B for 2 min. The total run time was 13 min. The diverter valve on the MS was set to send the elute to waste from 0 to 5 min, to MS from 5 to 7 min, and then to waste from 7 to 13 min. An Agilent 6460C Triple Quadrupole mass spectrometer (Agilent Technologies INC.) was set to run in scan mode for compound identification, and MRM mode for tricin quantification. Nitrogen was used as sheath gas at a flow rate of 11 L/min at 350 °C. The capillary gas temperature was 300 °C, the flow rate 10 L/min and the Nebulizer gas pressure 45 psi. The ESI spray capillary voltage was 3,500 V in positive ionization mode and 3,500 V in negative ionization mode. The fragmentor voltage was 135 and the cell accelerator voltage was 7. A separate standard containing 0.04 mg/mL tricin and 0.04 mg/mL umbelliferone was run with samples to determine the response factor and quantify the tricin content in the samples. Processing of data was completed off-line using Agilent’s Masshunter qualitative data analysis software.

**THIOACIDOLYSIS PROCEDURE**

1. Accurately weigh 2.5 mg of dry ground biomass into a 2 mL Micro-Reaction Vessel.

2. Pre-heat the MULTIVAP high temperature dry block to 100 °C.

3. Add 1.0 mL of thioacidolysis reagent using an Advanced Single Syringe Dispenser.

4. Cap the vials tightly.

5. Put the vials into the position holes of the high temperature dry block. Set the reaction time for 4 hours and start the timer.

6. Briefly vortex the vials every hour.

7. After 4 hours of reaction, move the vials to the vial rack and let the samples cool down to room temperature. Reset the MULTIVAP high temperature dry block to 55 °C.

8. For lignin analysis: To 4 mL glass vials placed in a vial rack, add 190 μL of saturated NaHCO_3_ solution. Transfer 400 μL of solution from the reaction vials into the 4 mL glass vials by pipetting and mix the solution by pipetting up-and-down twice.

9. For tricin analysis: To a second set of 4 mL glass vials, add 190 μL of saturated NaHCO_3_ solution. Transfer 400 μL of solution from the reaction vials into the 4 mL glass vials by pipetting and mix the solution by pipetting up-and-down twice.

10. Place the 4 mL glass vials in the dry block, turn on the nitrogen gas and adjust the nitrogen gas flow with the adjustable flow meter and pressure regulator. Wait until the samples have dried completely.

**DERIVATIZATION OF SAMPLES FOR GC-MS LIGNIN ANALYSIS**

1. To the dried samples, add 200 μL of derivatization reagent. Screw the caps on tightly and vigorously vortex the vials for 10 seconds.

2. Place the glass vials back in the heating dry block and keep them at 55 °C for 30 min.

3. Vigorously vortex the glass vials for 10 seconds, place them into a rack and let them cool to room temperature.

4. Transfer 100 μL of sample to a 2 mL GC autosampler vial with glass insert, tightly close the cap and the sample is ready for GC-MS analysis.

**RESUSPENSION OF SAMPLES FOR LC-MS TRICIN ANALYSIS**

1. To the dried samples, add 600 μL of 100% methanol, briefly vortex and place the samples in the 55 °C heating dry block for 15 min.

2. Vigorously vortex the glass vials for 10 seconds and place the samples in a vial rack.

3. Transfer 100 μL clear solution to a 2 mL GC autosampler vial with glass insert, tightly close the cap and the sample is ready for LC-MS analysis.
